# Emerging Seafood Preservation Techniques to Extend Freshness and Minimize *Vibrio* Contamination

**DOI:** 10.3389/fmicb.2016.00350

**Published:** 2016-03-22

**Authors:** Jennifer Ronholm, Fiona Lau, Swapan K. Banerjee

**Affiliations:** ^1^Microbiology Research Division, Bureau of Microbial Hazards, Food Directorate, Health Products and Food Branch, Health CanadaOttawa, ON, Canada; ^2^University of OttawaOttawa, ON, Canada

**Keywords:** *Vibrio*, seafood, shelf-life extension, irradiation, phage treatment, ozone treatment

## Abstract

Globally, the popularity of seafood consumption is increasing exponentially. To meet the demands of a growing market, the seafood industry has increasingly been innovating ways to keep their products fresh and safe while increasing production. Marine environments harbor several species of indigenous microorganisms, some of which, including *Vibrio* spp., may be harmful to humans, and all of which are part of the natural microbiota of the seafood. After harvest, seafood products are often shipped over large geographic distances, sometimes for prolonged periods, during which the food must stay fresh and pathogen proliferation must be minimized. Upon arrival there is often a strong desire, arising from both culinary and nutritional considerations, to consume seafood products raw, or minimally cooked. This supply chain along with popular preferences have increased challenges for the seafood industry. This has resulted in a desire to develop methodologies that reduce pathogenic and spoilage organisms in seafood items to comply with regulations and result in minimal changes to the taste, texture, and nutritional content of the final product. This mini-review discusses and compares several emerging technologies, such as treatment with plant derived natural compounds, phage lysis, high-pressure processing, and irradiation for their ability to control pathogenic vibrios, limit the growth of spoilage organisms, and keep the desired organoleptic properties of the seafood product intact.

## Introduction

Global seafood consumption has increased dramatically in the last few decades from an average of 9.9 kg per capita in the 1960s to 18.9 kg in 2010, this trend is expected to continue, putting additional pressure on our aquaculture systems (FAO, 2014). Post-harvest seafood harbors microorganisms acquired from the harvest site and some of these organisms can facilitate spoilage or be hazardous to human health. Seafood refers to mollusks (oysters, clams, and mussels), finfish, marine mammals, fish eggs (roe), and crustaceans (shrimp, crab, and lobster); however, some commodities are inherently more risky than others. As an example, there were 122 cases of *Vibrio parahaemolyticus* infections reported in western Canada between 2001 and 2006, 66.7% of infections were acquired through the consumption of raw oysters (Khaira and Galanis, 2007). Oysters pose the highest risk of infection for two reasons: oysters feed by filtering large volumes of seawater, during this process they may accumulate and concentrate pathogenic microorganisms that are naturally present in the water and for culinary reasons they are generally consumed without cooking. Therefore, oysters are a primary focus of novel intervention strategies.

Since the 1970s the bacterial pathogens primarily associated with illness due to seafood consumption have been from the genus *Vibrio*, specifically the species *V. cholerae*, *V. parahaemolyticus*, and *V. vulnificus* (DePaola et al., 2010). These vibrios occur naturally in marine environments, and with the exception of toxigenic *V. cholerae* O1, are not associated with fecal pollution (DePaola et al., 2010). Infection with *V. vulnificus* is rare; for example, in Canada only 2% of *Vibrio* infections (7 of 330 cases) were due to *V. vulnificus* between 2007 and 2013 (NESP, 2016). However, *V. vulnificus* infections can manifest as acute gastroenteritis, necrotizing wound infections, or invasive septicemia and have a mortality rate of approximately 50%, making it the leading cause of seafood associated mortality (Mead, 1999). Infection occurs mainly in individuals with pre-existing conditions who have consumed raw oysters (Nishibuchi and DePaola, 2005). While case fatality is lower, *V. parahaemolyticus* is the leading cause of acute gastroenteritis associated with the consumption of seafood; *V. parahaemolyticus* was responsible for 60% of *Vibrio* infections in the USA in 2013 (CDC, 2015), and 57% of *Vibrio* infections in Canada during 2007–2013 (NESP, 2016).

Several recent epidemiological studies have shown that general measures aimed at preventing environmental contamination and temperature control can be very effective at increasing shelf-life and reducing the prevalence of seafood-borne infections. For example, from 1999 to 2001, in response to a record number (12,318) of cases of *V. parahaemolyticus* in 1998, Japan released new regulations concerning how commercial enterprises handle seafood, including: the use of sterile or artificial seawater for washing, soaking, preserving, and cooling seafood after it has been boiled, maintaining fresh seafood at or below a temperature of 10°C, not consuming raw seafood with a *V. parahaemolyticus* level above 100 MPN/g, and consuming food within 2 h of being removed from refrigeration (Hara-Kudo and Kumagai, 2014). These control measures reduced infections rates by 99-fold the year after they were introduced (Hara-Kudo and Kumagai, 2014). In 2003, California instituted a regulation indicating that oysters to be consumed raw could not be harvested from the Gulf of Mexico during April 1–October 1, corresponding to the warmer high-risk season, in an effort to lower the incidence of *V. vulnificus* infection, which varied between 0 and 6 cases annually. After 2003 no further infections of *V. vulnificus* have been recorded in that state (Vugia et al., 2013).

**FIGURE 1:**
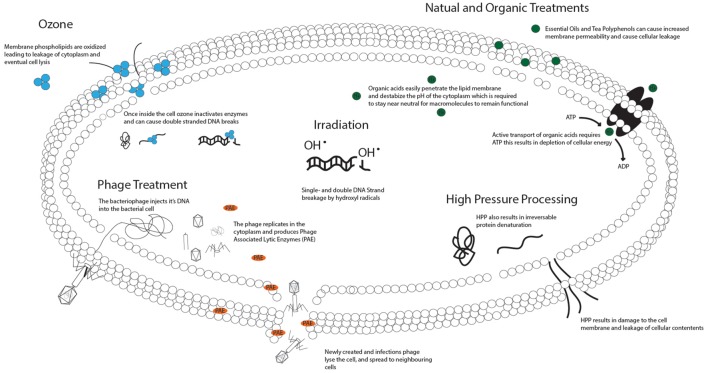
**Mechanisms of action of novel food safety interventions on bacterial cells.** Ozone acts by oxidizing various components of the cellular membrane leading to leakage of the cellular contents and eventually lysis (Dave, 1999). Once inside the cell ozone oxidizes proteins and enzymes resulting in a break-down of cellular function (Sykes, 1965) and single or double DNA strand breaks, eventually resulting in cell death (Hamelin, 1985). Phage treatment relies on the administration of a phage able to infect the target bacteria. The phage binds to the outside of the bacterial membrane and inserts its DNA into the host cell. The DNA encodes information to produce new viruses as well as Phage Associated Lytic Enzymes (PAE), which cause bacterial lysis (Trudil, 2015). Irradiation causes the radiolysis of water, resulting in production of the hydroxyl radical. The hydroxyl radical reacts with the sugar-phosphate backbone of DNA giving rise to single- and double-stranded DNA breaks. In small doses this leads to genomic mutations, in large doses it leads to chromosome breakdown and cellular death (Manas and Pagan, 2005). Bacterial inactivation by HPP results from the destruction of several cellular targets; however, the membrane, which has increased permeability after HPP, appears to be the primary target. In addition, at a certain pressure HPP irreversibly denatures proteins required for bacterial metabolism, together these changes result in death of the cell (Manas and Pagan, 2005). Natural Organic Treatments act on multiple targets within bacterial cells. Essential oils and Tea polyphenols have been shown to increase membrane permeability (Lambert et al., 2001; Daglia, 2012), and organic acids create an imbalance in the cytoplasmic pH resulting in loss of macromolecule functionality and depletion of cellular energy due to active export acids (Ricke, 2003).

The ultimate goal is to safely consume raw seafood, particularly oysters, from all harvest grounds year round. Therefore, novel and non-thermal technologies that are able to reduce pathogens, extend shelf-life, and preserve the nutritional and culinary benefits of the raw product, are strongly desired. Here, we review emerging technologies in terms of efficiency of treatment, mechanism of action, effects on the bacterial cell (**Figure 1**), effects on the food, and overall safety of use (**Table 1**).

**Table 1:** Principle, mechanisms of resistance, primary hazards, and the impact on food of each emerging technology.

	Principle	Impact on organoleptic seafood qualities	Mechanisms of bacterial resistance	Primary hazards
Ozone	Oxidizes biologically active macromolecules	– Neutral* – Does not oxidize seafood lipids (Chawla et al., 2007)	– None reported*	– GRAS – Residues of treatment include CO_2_ and H_2_O (Gonclaves, 2009)
Phage treatment	Bacteriophages infect and lyse bacterial cells	– Neutral*	– Blocking phage receptors – Production of extracellular matrix – Preventing DNA entry (Labrie et al., 2010)	– GRAS – Naturally found in most food products (Jun et al., 2014)
Natural organic treatments	Natural organic treatments change parameters of the food making it difficult for microbial survival and proliferation	– Variable	– Variable	– Not Hazardous – Naturally occurs in foods
High pressure processing	HPP damages cellular membranes, changes cell morphology, and denatures proteins	– Negative – Oyster meat becomes whiter and more opaque with increased pressure (Murchie et al., 2005) – Results in the cooked appearance fish (Master et al., 2000)	– Bacterial strains have high variability in resistance, the reasons are not understood*	– GRAS – Leaves no residue
Irradiation	Directly damages DNA of living organisms	– Neutral – No change in the sensory characteristics of oyster meat (Andrews et al., 2003; Jakabi et al., 2003; Song et al., 2009)	– Variable – Low resistance in food borne bacteria	– GRAS – Leaves no residue – Approved by several governments including the FDA, UK, and France (Wang et al., 2015) – Can be used on frozen food without the requirement of thawing


*Indicates that additional research is required in this area. GRAS – generally recognized as safe.

## Ozone Treatment

Ozone treatment, either by gaseous or dissolved forms, is among one of the most powerful oxidizing and food contact sanitizing treatments approved by the U.S. Food and Drug Administration (FDA). Ozone treatments oxidize various cellular components leading to membrane leakage and eventually cell death (**Figure 1**), it has high biocidal activity, requires short contact times, and can take place at the aquaculture level or on the final product. As an example of the former, applying 0.07 mg/L of ozone directly to seawater at shrimp hatcheries has been shown to allow the survival of shrimp, but eliminate pathogenic vibrios (Blogoslawski and Stewart, 2011). Studies on seafood spoilage are generally assessed based on several metrics including bacterial levels (CFU/g), the levels of gases produced from seafood breakdown such as total volatile basic nitrogen (TVB-N), trimethylamine nitrogen (TMA-N), total volatile acid (TVA), and ranking the food on fit-for-consumption scales based on sight and smell (Ordóñez et al., 2000; Arkoudelos et al., 2007; Pantazi et al., 2008). These methods are complementary, but each has a high-level of consensus when determining if a seafood product is spoiled (Pantazi et al., 2008). Most research in this area has focused on the ability of ozone to extend shelf-life. Ozone treatment of freshly harvested shrimp was evaluated for the ability to extend shelf-life using bacterial levels, TVB-N, TMA-N, and sensory characteristics as the evaluation criteria. After washing shrimp in ozone treated water for 1 min shelf-life was extended by 1.75 days (Okpala, 2014). Trout filets treated with ozone for two hours, were assessed by (TVB-N) measures, and found to have a shelf-life of 6 days, as compared to 4 days for untreated filets (Dehkordi and Zokaie, 2010). Shucked and vacuum-packaged mussels were assessed by spoilage bacteria levels, TVB-N, and sensory evaluation and were shown to have a shelf-life of 12 days after ozone treatment, as opposed to 9 days without treatment (Manousaridis et al., 2005).

Ozonised water can be used to produce slurry ice; however, the results of using this product to extend the shelf-life of seafood appear to depend on the nature of the seafood product. While, one study found that storage on ozonised slurry ice extended the shelf-life of sardines from 15 to 19 days (Campos et al., 2005), a more recent study found no increase in shelf-life of Tiger grouper, a reef fish, after storage on ozonised slurry ice (Karim et al., 2015).

## Natural Organic Treatments

Adding essential oils, tea polyphenols, and organic acids to seafood products has been suggested to extend shelf-life, limit pathogen proliferation, and maintain a synthetic preservative free marketing status. Essential oils such as thyme, oregano, rosemary, turmeric, and shallots have been shown to decrease the levels of non-pathogenic spoilage bacteria in seafood, when used in concentrations as low as 0.05 mg/mL (Harpaz et al., 2003; Pezeshk et al., 2011; Li et al., 2012). A variety of polyphenols including catechins, epigallocatechin gallate (EGCG), epigallocatechin, epicatechin gallate, and epicatechin, can be extracted from tea and have been shown to have antioxidant and antimicrobial properties (Fujiki, 1999). For example, immersing shrimp in a 0.01% catechin solution for only 15 min slowed the growth of spoilage bacteria, reduced the *Enterobacteriaceae* count, and had other quality enriching effects on the shrimp such as reducing lipid oxidation and melanosis (Nirmal and Benjakul, 2009). Dipping treatments of dried-seasoned jumbo squid in a mixed tea phenol solution also showed a protective effect against bacterial spoilage, moisture loss, oxidation of lipids, and degradation of lipids (Dong et al., 2013). Tea phenol treatment has also been shown to have a synergistic effect when combined with ozone treatment to extend shelf-life, and reduce nucleotide breakdown and lipid oxidation (Feng et al., 2012). Organic acids such as citric acid (300 mg/mL) and lactic acid (150 mg/mL) have been shown to reduce growth of spoilage organisms in freshly shucked oyster sample; in addition, dipping treatments of oysters in each of these organic acids showed a reduction of potentially pathogenic *V. vulnificus* below the detection level of 1.0 log/g from an initial artificially inoculated concentration of 6.0 log/g (Mahmoud, 2013).

## Phage Treatment

Two different phage groups have shown promise in controlling populations of *V. parahaemolyticus* in raw oysters: a Siphoviridae phage pVp-1 (Jun et al., 2014), and VPp1a phage isolated from *V. parahaemolyticus* (Peng et al., 2013). Depuration is a control process in which molluscs are held in potable water that has been treated with chlorine, ozone, or UV light, for a few hours before consumption to reduce their bacterial loads through the process of filter feeding. While this method is very effective in reducing coliform counts, unless it is carried out at low temperature and for a period of days, depuration is generally not effective against vibrios (Phuvasate et al., 2012). However, depuration in the presence of the phage VPp1a was able to reduce *V. parahaemolyticus* concentrations by 2.35–2.76 log CFU/g over a period of 36 h at 16°C (Rong et al., 2014). The phage pVp-1 was investigated for its ability to eliminate *V. parahaemolyticus* contamination as applied as both a bath immersion and directly to contaminated oyster meat. Both application strategies resulted in decreases in *V. parahaemolyticus* population, bath immersion treatment reduced the bacterial counts from 8.9 × 10^6^ CFU/g to 14 CFU/g after 72 h; however, direct application of phage to contaminated meat almost eliminated contamination within 12 h at 18°C, with 1.4 × 10^6^ CFU/g in the control and just 1.9 CFU/g in the treated samples (Jun et al., 2014). However, shellfish presents several challenges for phage treatment, the large and uneven surface area, when applied on in-shell oysters, limits contact time between phage particles and bacterial targets (Guenther et al., 2009).

## High Pressure Processing

High pressure processing (HPP), is commercially used at a range between 200 and 600 MPa, as an alternative to thermal processing (Considine et al., 2008). HPP treatment as short as 1–2 min on oysters increases shelf-life by as many as 11 days, by lowering the overall bacterial load and in the process kills the oyster causing the adductor muscle to release and making shucking easier (He et al., 2002). Particular attention has been paid to the ability of HPP to reduce *V. parahaemolyticus* and *V. vulnificus* in oysters. At harvest, the density of *V. parahaemolyticus* is generally less than 10^3^ MPN/g; however, pathogen levels rapidly increase to >10^6^ MPN/g if the storage temperature is not properly controlled (Gooch et al., 2001), and these levels are hazardous to human health. Considerable differences in pressure resistance between *V. parahaemolyticus* strains have been reported (Kural, 2008) making guidelines for effective HPP treatments to remove *V. parahaemolyticus* difficult. Differences of 1→7 log reductions in *V. parahaemolyticus* have been reported, based on several variables including: pressure level, suspension medium, processing time, processing temperature, and whether a whole shelled oyster or just meat tissue is assayed (Calik et al., 2006; Kural, 2008; Phuvasate and Su, 2015). While increasing pressure and processing time increase the log-reductions observed for *V. parahaemolyticus*, HPP appears to be more effective against *V. parahaemolyticus* when carried out at lower temperatures (Phuvasate and Su, 2015). If the temperature is lowered to 1.5°C from 20°C, the processing time can be lowered from 10 to 5 min and the pressure can be lowered to 250 MPa from 300 MPa without a loss in log reduction (Phuvasate and Su, 2015). Storage temperatures of seafood prior to HPP do not appear to affect resistance of *V. parahaemolyticus* to HPP; however, cold storage may increase the resistance of *V. vulnificus* to HPP treatment by increasing the percentage of polyunsaturated fatty acid in the cell membrane (Ye et al., 2013). Post-HPP cold storage has been shown to cause additional reductions in cell number after HPP-treatment, which may be occurring due to inhibition of recovery of sub-lethally injured cells (Ye et al., 2013). Despite its advantages, HPP also leads to meat becoming more opaque (Murchie et al., 2005), and results in the cooked appearance of several types of fish (Master et al., 2000), two factors that may limit its acceptance by consumers (**Table 1**).

## Irradiation

Irradiation of food products has become an emerging technology with promising features to enhance the safety and shelf-life of many different food types. Irradiation offers several unique characteristics including direct inactivation of organisms in frozen foods (Farkas, 1998). The use of gamma irradiation and more recently X-rays to eliminate pathogenic strains of bacteria such as vibrios in live oysters is becoming a popular alternative to thermal treatment (Grodner and Andrews, 1996; Farkas, 1998; Andrews et al., 2003; Jakabi et al., 2003; Mahmoud, 2009).

Gamma irradiation dose levels from 0.5–3.0 kGy have been tested on live oysters with studies showing that the maximum dose of 3.0 kGy did not kill the oysters or affect any of their sensory attributes. Although, reductions of 6-log *V. parahaemolyticus* were observed when dosage levels as low as 1.0 kGy were used (Jakabi et al., 2003). X-ray treatments on laboratory inoculated *V. parahaemolyticus* ready to eat shrimp products treated with 0.1–4 kGy X-ray levels showed a 6-log reduction in CFUs at 3 kGy (Mahmoud, 2009). To achieve a 6-log reduction of *V. vulnificus* in oysters 1.0 kGy was required for half shell oysters and 3.0 kGy for whole shell oysters (Mahmoud, 2009).

## Conclusion

Here, we have presented a short overview of several novel seafood preservation interventions along with the effect they have on bacterial cells (**Figure 1**), seafood quality, and possible hazards (**Table 1**). None of the technologies reviewed here represents a hazard in the food supply. However, some have unique advantages: the effectiveness of irradiation on frozen food without the need for thawing prior to treatment, and disadvantages: the bleaching of oyster meat exposed to HPP. Based on the variability of the effectiveness of the intervention, and the effects of the intervention on the seafood product we note the importance of validation of each sanitization strategy on each seafood product prior to routine use.

## Author Contributions

All authors listed, have made substantial, direct and intellectual contribution to the work, and approved it for publication.

## Conflict of Interest Statement

The authors declare that the research was conducted in the absence of any commercial or financial relationships that could be construed as a potential conflict of interest.
